# Novel H7N2 and H5N6 Avian Influenza A Viruses in Sentinel Chickens: A Sentinel Chicken Surveillance Study

**DOI:** 10.3389/fmicb.2016.01766

**Published:** 2016-11-16

**Authors:** Teng Zhao, Yan-Hua Qian, Shan-Hui Chen, Guo-Lin Wang, Meng-Na Wu, Yong Huang, Guang-Yuan Ma, Li-Qun Fang, Gregory C. Gray, Bing Lu, Yi-Gang Tong, Mai-Juan Ma, Wu-Chun Cao

**Affiliations:** ^1^State Key Laboratory of Pathogen and Biosecurity, Beijing Institute of Microbiology and EpidemiologyBeijing, China; ^2^Wuxi Center for Disease Control and PreventionWuxi, China; ^3^Division of Infectious Diseases, Global Health Institute, Nicholas School of the Environment, Duke University, Duke University Medical CenterDurham, NC, USA

**Keywords:** avian influenza A virus, H7N2 virus, H9N2 virus, H5N6 virus, sentinel chicken, transmission

## Abstract

In 2014, a sentinel chicken surveillance for avian influenza viruses was conducted in aquatic bird habitat near Wuxi City, Jiangsu Province, China. Two H7N2, one H5N6, and two H9N2 viruses were isolated. Sequence analysis revealed that the H7N2 virus is a novel reassortant of H7N9 and H9N2 viruses and H5N6 virus is a reassortant of H5N1 clade 2.3.4 and H6N6 viruses. Substitutions V186 and L226 (H3 numbering) in the hemagglutinin (HA) gene protein was found in two H7N2 viruses but not in the H5N6 virus. Two A138 and A160 mutations were identified in the HA gene protein of all three viruses but a P128 mutation was only observed in the H5N6 virus. A deletion of 3 and 11 amino acids in the neuraminidase stalk region was found in two H7N2 and H5N6 viruses, respectively. Moreover, a mutation of N31 in M2 protein was observed in both two H7N2 viruses. High similarity of these isolated viruses to viruses previously identified among poultry and humans, suggests that peridomestic aquatic birds may play a role in sustaining novel virus transmission. Therefore, continued surveillance is needed to monitor these avian influenza viruses in wild bird and domestic poultry that may pose a threat to poultry and human health.

## Introduction

Since the first emergence of avian influenza A(H7N9) virus in early 2013 (Gao et al., 2013), as of January 24, 2016, China has experienced four epidemic waves resulting in 711 laboratory-confirmed human infections with 283 deaths (Chinese National Influenza Center, 2016). The continuing circulation and evolution of H7N9 viruses in poultry (Lam et al., 2015), and the increasing number of human infections clearly identify H7N9 virus as an ongoing public health threat.

Domestic poultry are considered the main reservoir for H7N9 virus that cause human infections (Yu et al., 2013). However, wild birds, such as tree sparrows, songbirds, parakeets, and finches, have also been implicated as a source of virus transmission (Jones et al., 2014, 2015; Zhao et al., 2014). Because these wild birds share common space and resources with wild migratory birds, poultry, and humans, it is conceivable that wild avian species, especially aquatic birds, songbirds, passerine, and other small terrestrial birds might be infected with H7N9 virus and serve as vectors for dissemination of the virus to domestic poultry. However, research regarding H7N9 virus transmission from wild birds to poultry or vice versa has been sparse. Experimental studies of H7N9 virus natural transmission from wild avian birds to poultry may be necessary to examine such hypotheses. In view of this, surveillance of sentinel chicken for avian influenza viruses in a peridomestic aquatic areas (Coman et al., 2014) was undertaken to study transmission of H7N9 and other subtypes of avian influenza viruses.

## Materials and methods

### Study site

**Figure 1:**
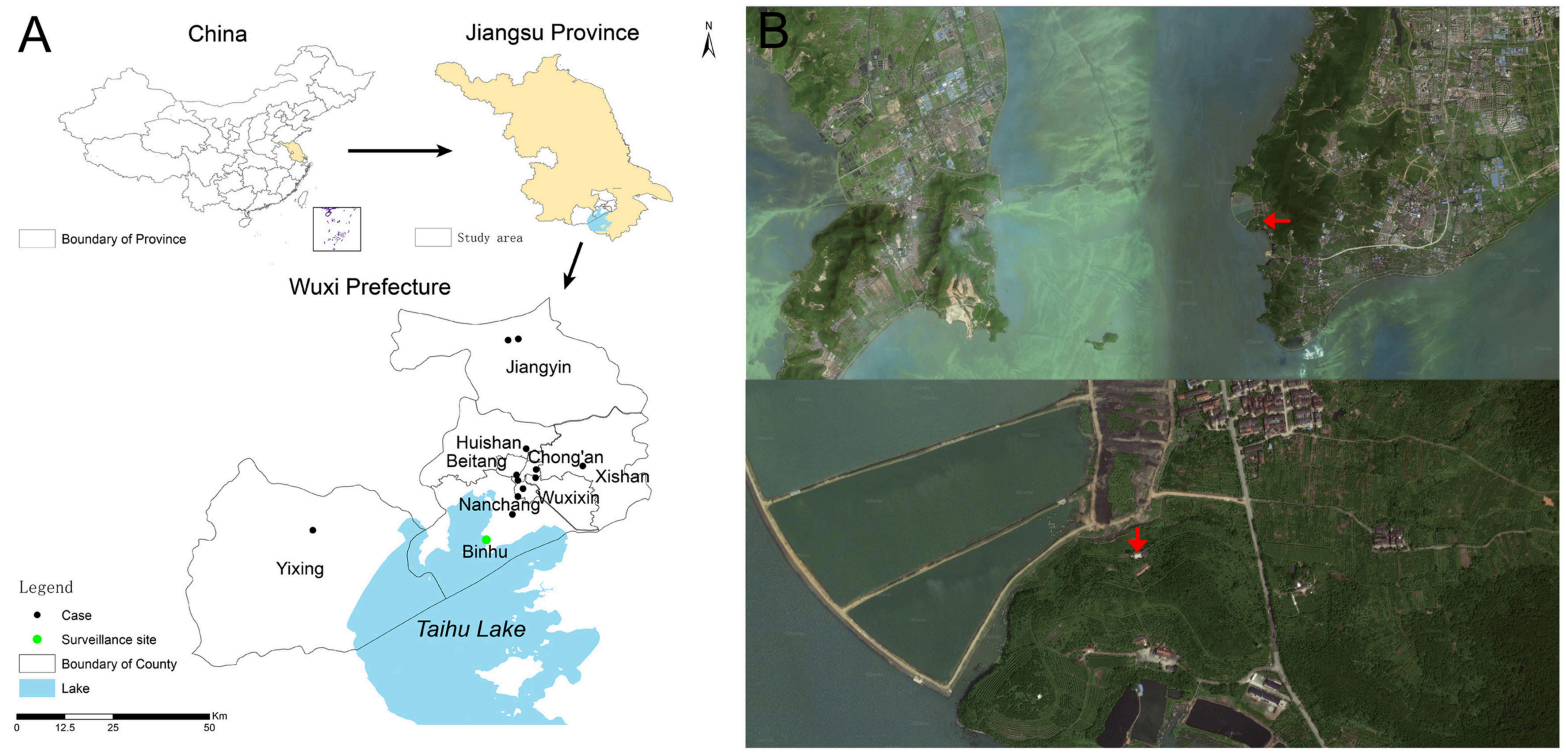
**Sentinel surveillance study site, Wuxi City, Jiangsu Province of China. (A)** Location of surveillance study site and distribution of human infections with influenza A (H7N9) virus; **(B)** Satellite map of study site, red arrows indicate study site.

During the period from January to October of 2014, a sentinel chicken surveillance study was conducted in Taihu Lake (Figure 1A) in Wuxi City, Jiangsu Province, China, where 13 human infections with H7N9 virus have been reported. Taihu Lake (30°55′40″–31°32′58″ N, 119°52′32″–120°36′10″ E) is China's third largest freshwater lake with an area of about 2338 km^2^. It is located in the Yangtze River delta and crosses Wuxi City, where 40 species of wild birds and 19 species of resident birds can be found and most of birds are winter migration birds (Zhao et al., 1990). These mainly include little egrets, herons, green-backed herons, black-crowned night herons, sparrows, quails, Chinese hwamei, yellow-browed warblers, Eurasian magpies, azure-winged magpies, and house swallows. The sentinel site was located on a foothill at the foot of the Lake's Mashan Mountain (Figure 1B) and was about 100 m^2^ in area such that the sentinel chickens would be free to move.

### Sentinel chickens and sample collection

We employed chickens as the sentinel bird for surveillance of avian influenza virus because chicken was one of main reservoir of H7N9 virus. In addition, influenza A virus negative chickens were easier to obtain than other domestic poultry such as ducks. A chicken sample size of 126 was chosen in order to detect a 10% avian influenza virus prevalence with a 95% confidence interval of ±5% with 80% power. However, a total of 135 chickens were used considering the death of chickens during the surveillance. On January 6, 2014, coincided with wild bird migration into the Taihu Lake, a total of 135 6-month-old, specific-pathogen-free (SPF) white leghorn chickens (Beijing Merial Vital Laboratory Animal Technology Co., Ltd., Beijing, China) were placed at sentinel surveillance site. A high rate of females to males (4:1) was chosen to avoid fighting among roosters. Before the sentinel chickens were placed into the surveillance site, cloacal and throat swabs of each chicken were collected, pooled, and screened for influenza A virus using a real-time RT-PCR assay which targeted the matrix gene (World Health Organization, 2014). In addition, an electronica ring (Beijing Raybaca Technology Co., Ltd) was fixed at the one leg of each chicken to identify their unique identity. The chickens were cared for and fed by a local farmer who agreed to assist us in this surveillance study. Each evening the chickens were encouraged with food to return to their protective enclosure.

During the surveillance period, cloacal and throat swabs from each chicken were collected and pooled together as one swab sample each month from February 6 to April 3 and from July 31 to August 20, and weekly from April 9 to June 26 as the number of wild birds increased in number due to in-migrations. All samples were placed in transport medium consisting of phosphate-buffered saline (PBS) containing 50% glycerol, penicillin (2000 U/ml), gentamicin (250 mg/ml), polymixin B (2000 U/ml), nystatin (500 U/ml), ofloxacin HCl (60 mg/ml), and sulfamethoxazole (200 mg/ ml) and were kept at 4°C for ~4 h until they were transported to the laboratory, where they were stored at −80°C for virus isolation within 2 days.

The chicken handling and sampling was performed in accordance with experimental animal administration and the ethics committee of Academy of Military of Medical Sciences. The protocol was approved by the biosafety committee of Beijing institute of Microbiology and Epidemiology.

### Virus isolation and sequencing analysis

All of swab samples were inoculated into the allantoic cavities of 9-day-old SPF embryonated chicken eggs (Beijing Merial Vital Laboratory Animal Technology Co., Ltd., Beijing, China) for virus isolation. Allantoic fluid were harvested after incubation at 37°C for 72 h or immediately after embryos died, and tested for hemagglutination (HA) activity with horse red blood cell and real time RT-PCR to detect the presence of influenza A virus. All virus isolation procedures were conducted in a biosafety level 3 facility. The whole viral genome of isolated viruses were sequenced by using Ion Torrent PGM sequencing technology (Life Technologies, Grand Island, NY, USA).

### Plaque purification of two co-infected samples

Two swab samples, #4315 and #6395, that contained mixed H7 and H9 subtypes were subjected to the standard plaque purification (Tobita et al., 1975) with a slight modification to segregate clones of individual viruses. Briefly, 100 μl aliquots of serial 10-fold dilutions of swab sample were inoculated into confluent monolayer MDCK cells cultured in 6 well-plates, MDCK cells were incubated at 37°C in a CO_2_ incubator for 1 h for virus absorption. Then covered with 1% agar containing MEM, penicillin (1000 unit/ml), streptomycin (1000 μg/ml), fungizone (2.5 μg/ml), MEM vitamin solution, 0.5% bovine serum albumin, and 2 μg/ml trypsin. After 2 days of incubation at 37°C in a CO_2_ incubator and stained with 0.025% neutral red containing 1% agarose, 42 and 53 plaques for #4315 and #6395 were initially picked for further purification and resuspended in 500 μl of infection medium. The subtypes of all resuspended virus stocks were verified using real time RT-PCR analysis (rRT-PCR), and virus stocks showing a single subtype were further purified in SPF chicken embryos. After three serial rounds of purification, the H7N2 and H9N2 virus isolates were successfully separated and their genome were fully sequenced. The sequences data generated in this study were deposited in Global Initiative on Sharing All Influenza Data (accession nos. EPI_ISL_223199–EPI_ISL_223202, EPI_ISL_223364). We used the maximum likelihood method to generate phylogenetic trees using MEGA version 6.0.6 (www.megasoftware.net/) with a bootstrapping resampling process (1000 replications) to assess the robustness of individual nodes of the phylogeny.

## Results

A total of 1623 pooled samples were collected from the sentinel chickens. In total, three (0.18%) of 1623 samples (samples #4315, #6395, and #7393) were positive for HA activity after virus isolation. Subtype determination, using rRT-PCR analysis targeting the H5, H7, and H9 HA genes, showed that specimens #4315 and #6395 were positive for all H7 and H9 genes, and specimen #7393 was only positive for H5 gene. To confirm these results, three HA-positive allantoic fluid specimens were subjected to sequencing on Ion Torrent PGM, and results consist with rRT-PCR that both specimens #4315 and #6395 that contained influenza A H7, H9, and N2 gene segments suggesting a coinfection of H7 and H9 subtypes, while specimens #7393 only presented with H5 and N6 type of influenza A virus. We then attempted to isolate individual H7 and H9 viruses from these two samples using a plaque purification. Fortunately, we were able to segregate the expected subtypes, H7N2 and H9N2 from two co-infected samples. Thus, totally five strains were isolated and designated as A/chicken/Wuxi/SC4315/2014 (H7N2), A/chicken/Wuxi/SC6395/2014 (H7N2), A/chicken/Wuxi/SC4315/2014 (H9N2), A/chicken/Wuxi/SC6395/2014 (H9N2), and A/chicken/Wuxi/SC7393/2014 (H5N6).

**Figure 2:**
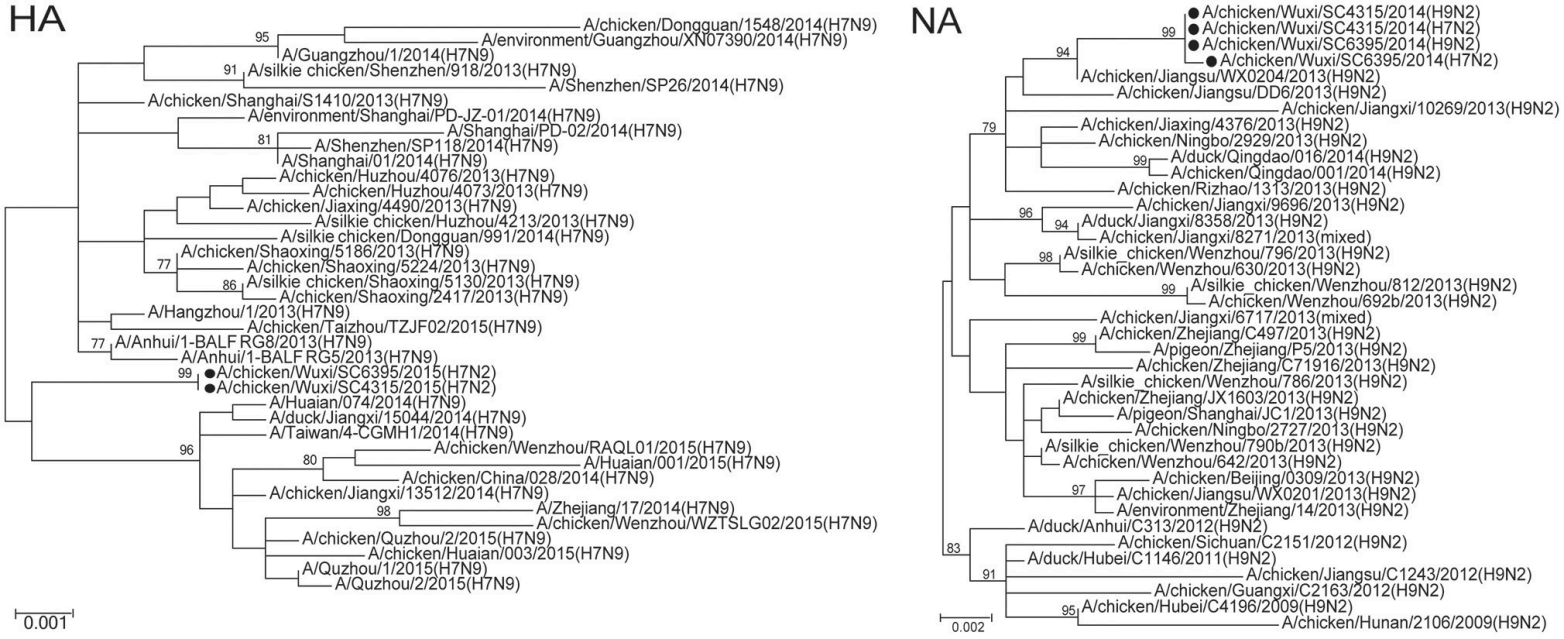
**Phylogenetic tree of hemagglutinin (HA) and neuraminidase (NA) genes of influenza A (H7N2) viruses isolated from sentinel chickens, Wuxi City, Jiangsu Province of China, 2014**. Supporting bootstrap values >75 are shown. The black circle dot indicates the viruses isolated in present study.

To characterize H7N2 viruses, molecular and phylogenetic analysis were conducted. The HA gene segments of the two H7N2 viruses most closely matched those of H7N9 viruses isolated from human and poultry in China in 2013 with an up to 99.6% nucleotide sequence identity (Table 1). Their NA segments were closely related to that of an H9N2 virus isolated from a chicken in Jiangsu Province, China during 2013 (99.5% identity). Their other six internal genes were derived from H9N2 viruses currently found in China with identities range from 99.5–99.7% (Table 1). In comparison with two H9N2 viruses in present study, all gene segments of two H7N2 viruses showed 99.0–100% total nucleotide sequence identity (Table 1). The two H7N9 viruses showed 100% total nucleotide sequence identity for HA, NP, NS, and PA, and >99.5% identity for other gene segments. Phylogenetic analysis based on HA genes showed that these two H7N2 viruses closely related with those H7N9 isolated in first and second waves, but were form an independent clade (bootstrap support of 99%) from a second H7N9 virus wave in China's Zhejiang, Jiangsu, and Jiangxi Provinces (Figure 2). Whereas, the other seven gene segments, including NA, clustered with those of the H9N2 viruses circulated in China and two H9N2 viruses identified in present study (Figure 2 and Supplementary Figure 1). These results suggest that the two H7N2 viruses are novel reassortant of H7N9 and H9N2 viruses recently circulated in China.

**Table 1:** **Influenza A viruses with greatest nucleotide sequence identity to avian influenza A (H7N2) viruses and sequence comparison of isolated in present study from Wuxi, China, 2014**.

**Gene**	**Virus designation**	**Identity, %**			
		**1**	**2**	**3**	**4**
**A/CHICKEN/WUXI/SC63951/2014 (H7N2)**					
HA	A/Anhui/1-BALF_RG8/2013(H7N9)	99.6	–	–	100.0
NA	A/chicken/Jiangsu/WX0204/2013(H9N2)	99.5	99.9	99.9	99.9
PB2	A/chicken/Beijing/16/2013(H9N2)	99.3	99.6	99.6	99.8
	A/chicken/Rizhao/55/2013(H9N2)				
PB1	A/chicken/Shandong/qd0917/2013(H9N2)	99.4	99.8	99.8	99.5
PA	A/chicken/Shandong/qd0917/2013(H9N2)	99.7	100.0	100.0	100.0
NP	A/chicken/Qingdao/2144/2013(H9N2)	99.5	99.9	99.9	100.0
M	A/chicken/Beijing/0331/2013(H9N2)	99.7	99.9	99.9	99.9
NS	A/chicken/Shandong/qd0917/2013(H9N2)	98.4	99.0	99.0	100.0
**A/CHICKEN/WUXI/SC43151/2014 (H7N2)**					
HA	A/Anhui/1-BALF_RG8/2013(H7N9)	99.6	–	–	–
NA	A/chicken/Jiangsu/WX0204/2013(H9N2)	99.6	100.0	100.0	–
PB2	A/chicken/Beijing/16/2013(H9N2)	99.3	99.6	99.6	–
	A/chicken/Rizhao/55/2013(H9N2)				
PB1	A/chicken/Shandong/qd0917/2013(H9N2)	98.9	99.2	99.2	–
PA	A/chicken/Shandong/qd0917/2013(H9N2)	99.7	100.0	100.0	–
NP	A/chicken/Qingdao/2144/2013(H9N2)	99.5	99.9	99.9	–
M	A/chicken/Beijing/0331/2013(H9N2)	99.8	100.0	100.0	–
NS	A/chicken/Shandong/qd0917/2013(H9N2)	98.4	99.0	99.0	–

*1, identity determined by a BLAST search of the Influenza Sequence Database; 2, identity compared with A/chicken/Wuxi/SC6395/2014(H9N2); 3, identity compared with A/chicken/Wuxi/SC4315/2014(H9N2); 4, identity compared each other*.

*HA, hemagglutinin; NA, neuraminidase; PB, polymerase basic; PA, polymerase basic; NP, nucleoprotein; M, matrix; NS, nonstructural*.

**Figure 3:**
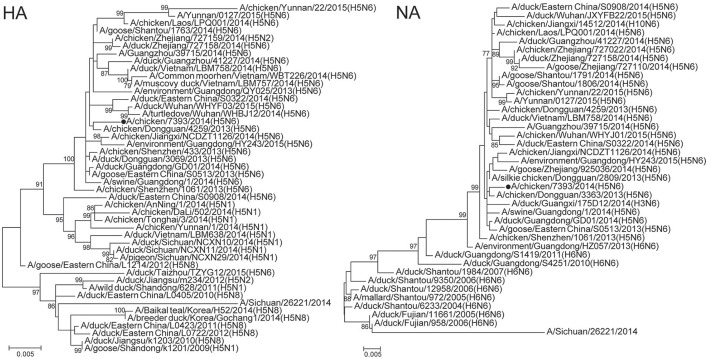
**Phylogenetic tree of hemagglutinin (HA) and neuraminidase (NA) genes of influenza A (H5N6) viruses isolated from sentinel chickens, Wuxi City, Jiangsu Province of China, 2014**. Supporting bootstrap values >75 are shown. The black circle dot indicates the viruses isolated in present study.

As for that H5N6 virus, the HA gene phylogeny showed that the virus was closely related to A/chicken/Dongguan/4259/2013(H5N6) clade 2.3.4.4 from Guangdong Province, China. The virus likely originated from highly pathogenic H5 avian influenza viruses with N1, N2, and N8 neuriminidases detected in poultry in China since 2010 and from viruses detected in Vietnam in 2014 (Figure 3). The NA gene segment of the H5N6 virus clustered into Jiangxi lineage and likely originated from H6N6 avian influenza viruses circulating in China's Fujian and Guangdong Provinces (Figure 3). The six internal genes of the H5N6 virus were found to cluster with H5N6 strains identified in China and Vietnam (Supplementary Figure 2).

Molecular analysis of critical and apparent amino acid residues that may be associated with adaptation of an avian virus to the mammalian host, virulence, and antiviral resistance of H7N2 and H5N6 viruses were conducted (Table 2). The HA gene protein of both two H7N2 viruses had a single basic amino acid (PEIPKGR↓G) at the cleavage site, indicating low pathogenic effects in poultry. Substitution at A138, A160, V186, and L226 (H3 numbering) was observed suggesting a possible increased affinity for binding human α2, 6-linked sialic acid receptors. Three amino acid deletions (63–65) were observed in the stalk region of NA protein. Although no substitutions of the H274K and R292K (N2 numbering) amino acid were observed, indicating that the viruses should still be sensitive to oseltamivir, zanamivir, and peramivir, mutation of V222 and K249 were observed in two viruses. Whereas, the HA gene protein of the H5N6 virus processed multiple basic amino acid motifs (RERRRKR↓G) at cleavage site, indicated highly pathogenic phenotype (Table 2). While the HA gene protein of H5N6 virus had the amino acids Q226 and G228 (H3 numbering) indicated that the virus preferentially bind to avian-like receptors (Stevens et al., 2006), but the mutation of P128, A138, and A160 may enhance binding capacity to human like receptors. The NA stalk of H5N6 virus possessed a deletion of 11 aa residues at positions 58–68 (N6 numbering), which suggested that the virus isolate might have different adaptation and virulence characteristics in poultry and mammals (Bi et al., 2015). Moreover, H5N6 virus contained a truncated PB1-F2 protein of 57 aa in length, which might influence their virulence in mammals (Zamarin et al., 2006). Amino acids V89, E627, and D701 were observed in the PB2 protein of H7N2 and H5N6 viruses, suggesting that these viruses were not yet fully adapted to infect mammals (Li et al., 2005). No drug resistance-associated mutations were observed in the H5N6 isolate but mutation N31 in M2 protein was found in both two H7N2 viruses. Other possible molecular markers for two subtype viruses that may associated with virulence are shown in Table 2.

**Table 2:** **Influenza A viruses isolated in present study and mutation analysis of critical amino acid residues associated with virulence, adaptation to mammals or antiviral resistance, Wuxi, China, 2014**.

	**A/chicken/Wuxi/SC6395/2014**	**A/chicken/Wuxi/SC4315/2014**	**A/chicken/Wuxi/SC7393/2014**	**Comments**
Status of chickens	Healthy	Healthy	Sick	
Collection date	May 7, 2014	June 19, 2014	August 20, 2014	
**HA**				
Cleavage	PEIPKGR↓G	PEIPKGR↓G	RERRRKR↓G	
S128P	S	S	**P**	Mammalian adaptation
A135T	A	A	V	
S138A	**A**	**A**	**A**	
N158D	N	N	N	
T160A	**A**	**A**	**A**	
G186V	**V**	**V**	N	
Q226L	**L**	**L**	Q	
G228S	G	G	G	
**NA**				
Deletion	63-65 (N2 numbering)	63-65 (N2 numbering)	58-68 (N6 numbering)	Enhance virus adaptation to domestic fowl
E119V	E	E	E	Oseltamivir resistance
D198N	D	D	**N**	
I222V	**V**	**V**	I	
H274Y	H	H	H	
R292K	R	R	R	
N294S	N	N	N	
Q136K	Q	Q	Q	Zanamivir resistance
D151E	D	D	D	
R224K	R	R	R	
R249K	**K**	**K**	**K**	
E276D	E	E	E	
S315N	S	S	K	
R371K	R	R	R	
**PB2**				
E158G	E	E	E	Increased the morbidity and mortality of H5 viruses in mice
T271A	T	T	T	Enhance polymerase activity in human cells
L89V	V	V	V	Mammalian host adaption and increased virulence in mice
E627K	E	E	E	
D701N	D	D	D	
**PB1**				
H99Y	H	H	H	H5 virus transmissible among ferrets
I368V	**V**	**V**	I	
**PB1-F2**				
S12Stop	S	S	S	Enhanced transmission and pathogenicity to human for pH1N1
N66S	N	N	n/a	Increased the virulence of an H5N1 virus
58–90 truncated	No	No	Yes	Increased virulence in mammals
**PA**				
T97I	T	T	T	Increased pathogenicity in mice of H5N2 virus
T85I	T	T	T	Involved host adaptation of pH1N1
G186S	G	G	G	
L336M	L	L	L	
P400L	P	P	P	Enhanced polymerase activity in human cells
M423I	V	V	**I**	
V476A	**A**	**A**	**A**	
T552S	T	T	T	
**NP**				
A184K	**K**	**K**	**K**	Increased replication in mammalian cells pathogenicity
N319K	N	N	N	
**M1**				
N30D	D	D	D	Increased pathogenicity in mice of H5N1
T215A	A	A	A	
**M2**				
S31N	**N**	**N**	S	Antiviral resistance (amantadine)
**NS1**				
P42S	S	S	S	Increased virulence in mice or in pigs
D92E	D	D	**E**	

*HA, hemagglutinin; NA, neuraminidase; PB, polymerase basic; PA, polymerase basic; NP, nucleoprotein; M, matrix; NS, nonstructural. Bold represents substation*.

## Discussion

**Figure 4:**
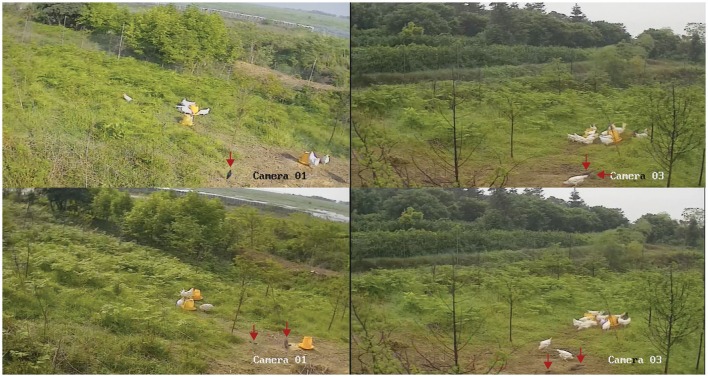
**Monitors captured partial image of birds during the surveillance period**. Red arrows indicate observed birds in study site.

We identified the novel H7N2 and H5N6 viruses during the surveillance of avian influenza virus using sentinel chickens in China in 2014. Although, we did not collect the samples from wild birds in local area because of restriction of regulations, our results suggest that the viruses were likely transmitted from wild birds to sentinel chickens in this aquatic environment because influenza A virus negative SPF chickens were used. However, we are uncertain which birds may have transmitted virus to the sentinel chickens or how that transmission occurred. While aquatic birds would be the prime transmission suspects, resident or passerine birds might also be transmitters (Jones et al., 2014). Avian influenza virus transmission could have occurred via another source such as contact with man, via bird feed, or contaminated drinking water (Jones et al., 2015). It is interesting that wild birds were observed mixing with sentinel chickens together, and they drank from the same water and ate the same bird feed (Figure 4). Therefore, most likely route for sentinel chickens infected with H7N2 and H5N6 viruses from wild bird is by means of drinking the water and eating the feed. However, the evidence for virus transmission via drinking water or eating the feed was limited because we did not collect these samples to test the presence of avian influenza virus.

Although, we did not find any H7N9 viruses among the sentinel chickens during the surveillance period, we identified the novel two H7N2 and one H5N6 viruses. Phylogenetic analysis revealed that the HA gene of H7N2 viruses were closely related to those of the H7N9 viruses, that emerged in China recently and the other seven gene segments (PB2, PB1, PA, NP, M, NS, and NA) were closely related to those of H9N2 viruses that were isolated from poultry in China, providing the evidence that H7N9 viruses continue to evolve and reassort with H9N2 viruses in poultry in China. Molecular analysis showed that the substitutions at A138, A160, V186, and L226 (H3 numbering) in HA protein were observed, which is proposed to enhance binding capacity of human like receptors and mammalian adaption. A deletion of three amino acids in the viral NA stalk has been observed. It has been suggested that amino acid deletion within the stalk domain may be associated with transmission of influenza from wild birds to domestic poultry (Cauldwell et al., 2014). However, it is unclear whether such H7N2 viruses would be also detected in wild birds because samples from wild bird were not collected in local. The viruses should still be sensitive to oseltamivir, zanamivir, and peramivir as no substitutions of the K274 and K292 amino acid were observed. Although, the single mutation of V222 or K249 may not be associated with marginal levels of oseltamivir resistance, increased drug resistance levels in association with other mutation (Simon et al., 2011). In addition, mutation of N31 in M1 protein suggests that the virus had the resistance to amantadine. Previous studies have showed that H7N9 isolates from humans with the mutation of glutamic acid to lysine at position 627 (E627K) in PB2 were much more efficient and more lethal in mice than H7N9 isolates from birds (Zhang et al., 2013, 2014). The H7N2 viruses identified in present study does not have the PB2 K627 and N701 mutations but had an V89 mutation suggests that the viruses have not yet fully adapted to infect mammals (Li et al., 2005). However, H7N2 virus identified by Shi et al. (2014) without K627 mutation but showed comparable replication ability in the lungs of mice with human H7N9 virus and a significantly higher replication than that of avian H7N9 virus. These findings suggest that the continued circulation of H7 viruses in nature will enable them to acquire more mutations or new gene constellations that might increase their virulence in animals or humans. Taken together these findings, the continued circulation of H7N9 and H9N2 viruses in poultry and persistent human infection of H7N9 virus, H7 viruses still pose seriously threats to human health, especially if they acquire mutations or new gene reassortments that increase their virulence in animals or humans.

Since the first H5N6 virus infections in human reported in China, a total of 15 patients have been reported. Previous studies have shown that H5N6 viruses isolated from the patients had similar identity with viruses isolated from the live poultry market and they are constantly evolving. In our study, we isolated a highly pathogenic avian influenza A (H5N6) virus that belong to clade 2.3.4.4 from a sentinel chicken. However, any death bird was not observed in study site during period of surveillance although H5N6 viruses have been detected in fatal wild birds (Yu et al., 2015). Sequence analysis revealed that the virus had a very closely genetic relationship with H5N6 viruses isolated from the patients and live poultry, thereby suggesting that direct contact with poultry contributed to the human infection. Furthermore, the H5N6 virus has distinct evolutionary characteristics, such as the P128, A138, and A160 mutations in the HA protein and an 11-aa deletion in the NA stalk, which may enhance their adaptation and infectivity in mammals, including humans. The virus may resistant to oseltamivir and zanamivir (N198 and K249) but sensitive to amantadine (S31). Other possible virulence molecular markers are shown in Table 2. Although the potential virulence mutations are described on the basis of previous studies in animals, the pathogenesis in humans remains unknown. Because the continue evolution of H5N6 viruses in human and poultry (Yuan et al., 2016), patients they had recent history of direct contact with poultry, and drug resistance mutation have been detected in viruses isolated from both human and poultry (Shen et al., 2016), the potential for infection, outbreaks, and pandemic in humans should be closely monitored.

The study had several limitations. Firstly, samples from wild birds were not collected in the study area due to the restriction of regulations. Secondly, environmental samples were not collected to test the presence of avian influenza viruses. Thirdly, the low virus isolation rate could be due to the possible low prevalence of avian influenza viruses and low viral load in samples.

In conclusion, our results suggest transmission of avian influenza viruses from wild birds to poultry. Hence, public health officials must consider methods to disrupt peridomestic novel avian influenza virus transmission in these aquatic bird environments.

## Author contributions

M-JM, TZ, Y-HQ, S-HC, G-LW, M-NW, YH, G-YM, L-QF, G-CG, BL, and Y-GT conducted the study and analyzed data, M-JM, S-HC, TZ, G-LW, M-NW, YH, and G-YM processed samples and virus isolation., M-JM and WC conceived and designed the study and wrote the manuscript.

### Conflict of interest statement

The authors declare that the research was conducted in the absence of any commercial or financial relationships that could be construed as a potential conflict of interest.

## Supplementary material

Supplementary Figure 1 **Phylogenetic relationships of internal genes of influenza A (H7N2) viruses from sentinel chickens, Wuxi City, Jiangsu Province, China, 2014**. Supporting bootstrap values >75 are shown. Red font indicates viruses isolated in present study. PB, polymerase basic; PA, polymerase acidic; NP, nucleoprotein; MP, matrix protein; NS, nonstructural. The black circle dot indicates the viruses isolated in present study.

Supplementary Figure 2 **Phylogenetic relationships of internal genes of influenza A (H5N6) viruses from sentinel chickens, Wuxi City, Jiangsu Province, China, 2014**. Supporting bootstrap values >75 are shown. Red font indicates viruses isolated in present study. PB, polymerase basic; PA, polymerase acidic; NP, nucleoprotein; MP, matrix protein; NS, nonstructural. The black circle dot indicates the viruses isolated in present study.
