# Supplementary figures and images for: Novel H7N2 and H5N6 Avian Influenza A Viruses in Sentinel Chickens: A Sentinel Chicken Surveillance Study
Source: Front Microbiol. 2016 Nov 16;7:1766. doi: 10.3389/fmicb.2016.01766 (PMC5110548; doi:10.3389/fmicb.2016.01766)

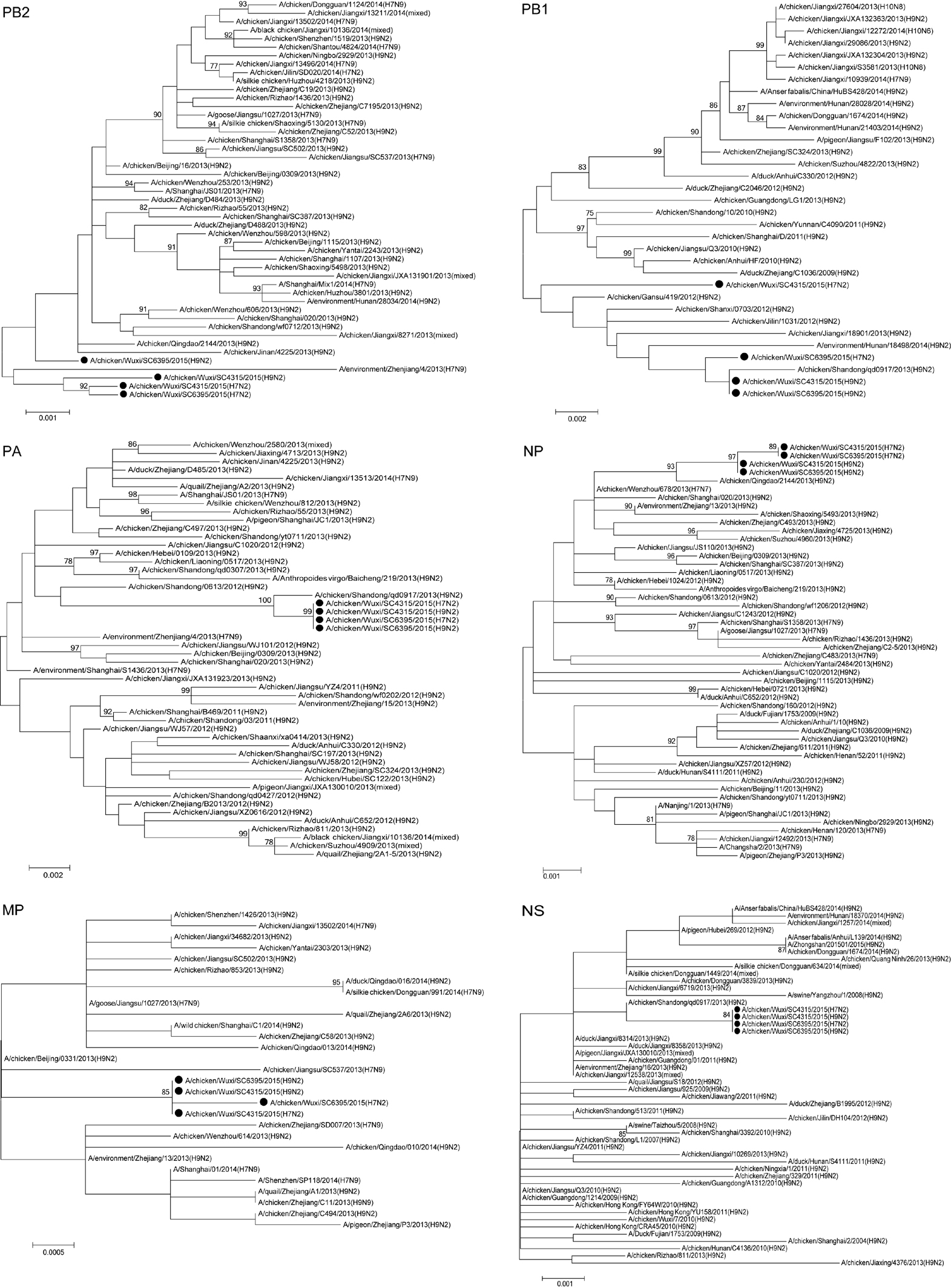

Supplement: Supplementary Figure 1 — Phylogenetic relationships of internal genes of influenza A (H7N2) viruses from sentinel chickens, Wuxi City, Jiangsu Province, China, 2014. Supporting bootstrap values >75 are shown. Red font indicates viruses isolated in present study. PB, polymerase basic; PA, polymerase acidic; NP, nucleoprotein; MP, matrix protein; NS, nonstructural. The black circle dot indicates the viruses isolated in present study. [file Image1.JPEG]

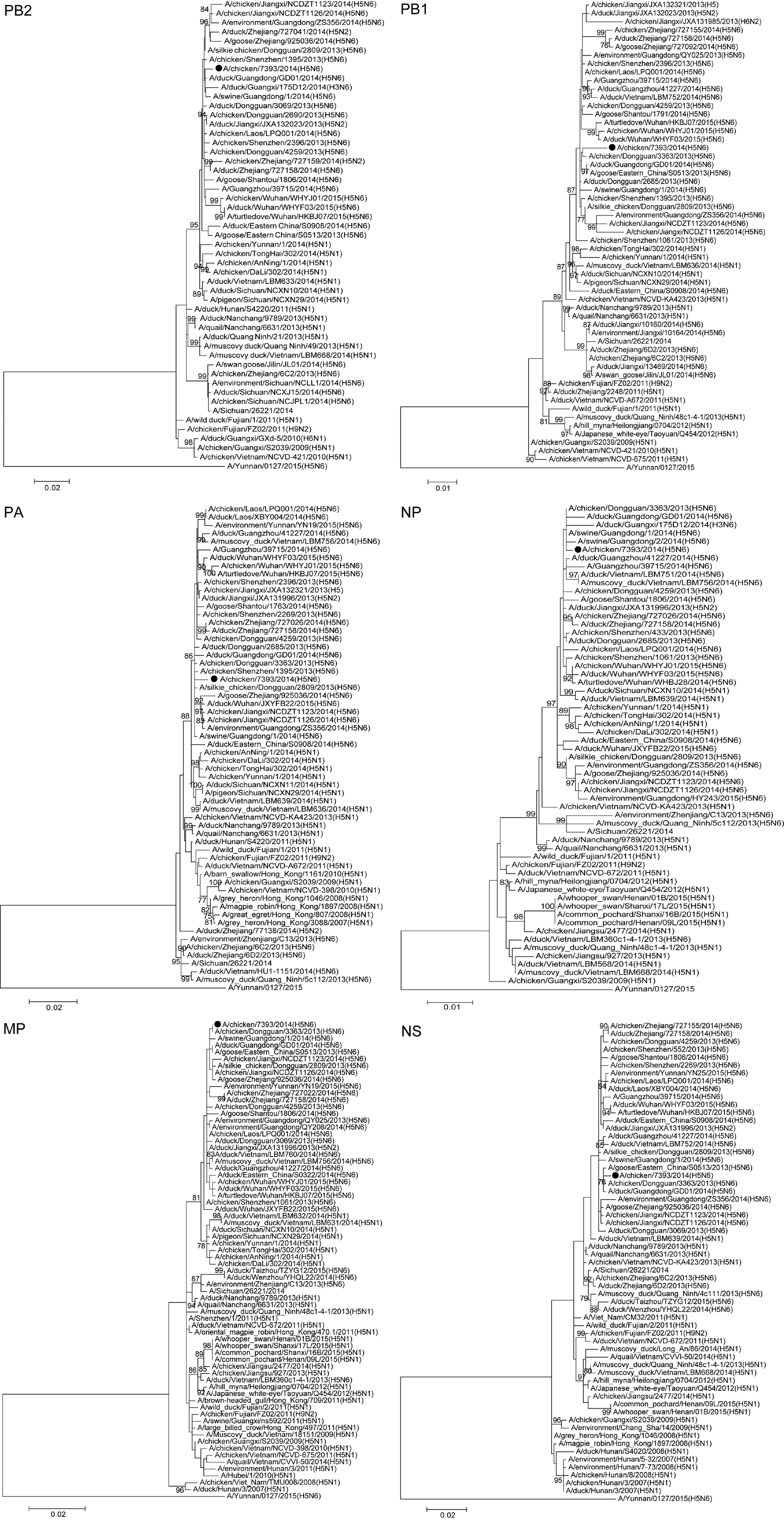

Supplement: Supplementary Figure 2 — Phylogenetic relationships of internal genes of influenza A (H5N6) viruses from sentinel chickens, Wuxi City, Jiangsu Province, China, 2014. Supporting bootstrap values >75 are shown. Red font indicates viruses isolated in present study. PB, polymerase basic; PA, polymerase acidic; NP, nucleoprotein; MP, matrix protein; NS, nonstructural. The black circle dot indicates the viruses isolated in present study. [file Image2.JPEG]
